# Coilin Phosphomutants Disrupt Cajal Body Formation, Reduce Cell Proliferation and Produce a Distinct Coilin Degradation Product

**DOI:** 10.1371/journal.pone.0025743

**Published:** 2011-10-03

**Authors:** Zunamys I. Carrero, Venkatramreddy Velma, Heather E. Douglas, Michael D. Hebert

**Affiliations:** Department of Biochemistry, The University of Mississippi Medical Center, Jackson, Mississippi, United States of America; Brunel University, United Kingdom

## Abstract

Coilin is a nuclear phosphoprotein that accumulates in Cajal bodies (CBs). CBs participate in ribonucleoprotein and telomerase biogenesis, and are often found in cells with high transcriptional demands such as neuronal and cancer cells, but can also be observed less frequently in other cell types such as fibroblasts. Many proteins enriched within the CB are phosphorylated, but it is not clear what role this modification has on the activity of these proteins in the CB. Coilin is considered to be the CB marker protein and is essential for proper CB formation and composition in mammalian cells. In order to characterize the role of coilin phosphorylation on CB formation, we evaluated various coilin phosphomutants using transient expression. Additionally, we generated inducible coilin phosphomutant cell lines that, when used in combination with endogenous coilin knockdown, allow for the expression of the phosphomutants at physiological levels. Transient expression of all coilin phosphomutants except the phosphonull mutant (OFF) significantly reduces proliferation. Interestingly, a stable cell line induced to express the coilin S489D phosphomutant displays nucleolar accumulation of the mutant and generates a N-terminal degradation product; neither of which is observed upon transient expression. A N-terminal degradation product and nucleolar localization are also observed in a stable cell line induced to express a coilin phosphonull mutant (OFF). The nucleolar localization of the S489D and OFF coilin mutants observed in the stable cell lines is decreased when endogenous coilin is reduced. Furthermore, all the phosphomutant cells lines show a significant reduction in CB formation when compared to wild-type after endogenous coilin knockdown. Cell proliferation studies on these lines reveal that only wild-type coilin and the OFF mutant are sufficient to rescue the reduction in proliferation associated with endogenous coilin depletion. These results emphasize the role of coilin phosphorylation in the formation and activity of CBs.

## Introduction

The Cajal body (CB) is a subnuclear structure that participates in the biogenesis of telomerase and ribonucleoproteins [1], [2]. Several proteins enriched within the CB are posttranslationally modified by phosphorylation [3]. Among these is coilin, which is considered the marker protein for CBs. Coilin, a protein of 576 amino acids in human, has at least 17 phosphorylated residues as identified using high throughput tandem MS/MS analyses [4], [5], [6], [7], [8], [9]. Coilin is necessary for proper CB formation, composition and activity, as evidenced by knockout and knockdown studies [10], [11], [12], [13]. Coilin knockdown in HeLa cells has been shown to reduce cellular proliferation [14], [15], presumably due to depleted small nuclear ribonucleoprotein (snRNP) resources.

CBs are most frequently detected in cells with high transcriptional demands, such as neuronal and cancer cells, but can also be observed, albeit less often, in other cell types such as fibroblasts [1], [2]. We have shown that coilin in primary cells that lack CBs appears to be more phosphorylated compared to that found in transformed cells that have CBs [16]. Additionally, the phosphorylation of coilin has been shown to increase during mitosis when CBs disassemble [16], [17]. This disassembly of coilin during mitosis is correlated with a reduction in coilin self-association [18].

**Figure 1:**
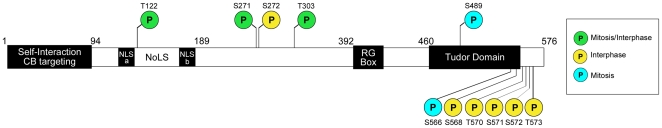
Schematic representation of human coilin showing the locations of the coilin self-interaction domain and apparent nucleolar localization signal (NoLS) [**18**], nuclear localization signals (NLS), RG box [**21**] and tudor domain [**19**]. Also indicated are 11 residues that are phosphorylated. Blue represents phosphorylation sites enriched during mitosis, yellow corresponds to those identified during interphase, and the phosphoresidues identified in both mitosis and interphase are green [9].

Little structural information on coilin exists, but a recent study has found that coilin contains a tudor-like domain between aa 460 and 560 [19] (Figure 1). The survivor of motor neuron protein, SMN, which is mutated in most cases of the neurodegenerative disease spinal muscular atrophy [20], also contains a tudor domain. The SMN tudor domain associates with symmetrically dimethylated arginine residues found on Sm proteins during snRNP biogenesis [1], [2], [3]. In contrast, the tudor domain of coilin contains extensive loops and does not appear to interact with methylated arginines [19], suggesting a different function for the coilin tudor domain compared to the SMN tudor domain. Interestingly, coilin contains a region (the RG box) N-terminal to the tudor domain (Figure 1) that contains symmetrically dimethylated arginines, and these residues influence the interaction of coilin with SMN [21], [22], [23], [24]. Since the C-terminus of coilin also mediates interaction with Sm proteins [21], [22], [24], and SMN and Sm proteins compete for binding sites on coilin [21], it is possible that the coilin RG box and tudor domain are necessary for the displacement of nascent snRNPs from the SMN complex during the Cajal body phase of snRNP biogenesis. Further support for the role of methylation on coilin interaction with SMN, comes from studies showing that hypomethylated coilin correlates with the presence of Gems, which are subnuclear domains that lack coilin but contain SMN and associated Gemins [1], [22], [23]. Methylation of coilin alone is not the only factor that contributes towards Gem formation, however, as other studies have shown that the level of SMN impacts Gem formation [25]. Besides its role in SMN interaction, coilin methylation also regulates the localization of this protein to the nucleolus [26]. In addition to methylation, phosphorylation of coilin also appears to impact its ability to interact with SMN and Sm proteins [9]. Specifically, SMN preferentially binds to hypophosphorylated coilin but SmB′ binds more to phosphorylated coilin.

Other studies have shown that coilin interacts with Ku proteins and can inhibit in vitro non-homologous DNA end joining [27] as well as influence cell viability in response to DNA damage caused by cisplatin [28]. Additionally, coilin has been shown to accumulate at centromeres damaged in response to herpes simplex type 1 infection [29] and form micro-foci after UV-C exposure [30]. These studies suggest that nucleoplasmic coilin, where the majority of the protein is found [31], may have a role in stress response pathways such as those caused by DNA damage. How phosphorylation of coilin impacts its putative role in these stress response pathways is unknown.

In order to better clarify the role of phosphorylation on CB formation, we utilized coilin phosphomutants expressed both transiently and stably after induction in HeLa cells. We examined proliferation rates in these cells and monitored CB formation both with normal and reduced levels of endogenous coilin. We have found that certain coilin phosphomutants inhibit cell proliferation while others have no effect, and this inhibition is associated with reduced CB number. Interestingly, two phosphomutants are degraded to an N-terminal fragment when expressed at levels close to that of the endogenous coilin, indicating a specific pathway for coilin degradation. These data demonstrate a crucial role for coilin phosphorylation in the formation of CBs.

## Results

### Transiently expressed coilin phosphomutants reduce cell proliferation

**Figure 2:**
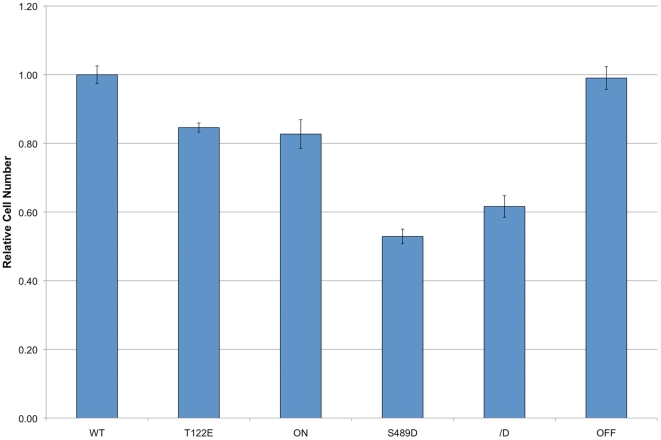
Transiently transfected coilin phosphomutants decrease the proliferation rate of HeLa cells. HeLa cells were transfected with GFP-coilin (WT) or GFP-coilin phosphomutants (T122E, ON, S489D, S271/272D or OFF). The proliferation rates were measured by conducting a cell titer blue assay 24 h, 48 h, and 72 h post transfection. The values for each transfectant obtained at 72 h were divided by the value of that line at 24 h. All lines were then normalized to WT. Proliferation rates were significantly reduced (p value<0.005) by the expression of all coilin phosphomutants except OFF. Errors bars denote the percent standard error based on an n of no fewer than 6.

Previous results have demonstrated that coilin reduction inhibits cell proliferation [14], [15]. Since coilin is a phosphoprotein whose phosphorylation increases during mitosis [17], we would expect that any phosphomutant that alters CB formation or activity would negatively impact proliferation. To test for this possibility, we transiently transfected HeLa cells with various GFP-tagged coilin phosphomimic and phosphonull constructs in order to examine if any of the phosphomutants acted in a dominant negative manner over endogenous coilin. These constructs include a wild-type sequence (denoted as WT) as well as mutations changing 11 of the known phosphorylated residues to aspartic or glutamic acid (denoted as ON) or alanine (OFF). Additionally, three other constructs were used: T122 was mutated to glutamic acid (T122E), S489 was mutated to aspartic acid (S489D) and S271/S272 were converted to aspartic acid (S271/272D) (Figure 1). T122 and S271/272 were selected for mutation because MS/MS analysis have demonstrated that these residues are phosphorylated in both interphase and mitosis [5], suggesting an essential role for these modifications in coilin activity throughout the cell cycle. In contrast, S489 was selected for mutation because the phosphorylation of this residue appears to be enriched during mitosis [5] when CBs are disassembled. We have previously shown that GFP-coilin (WT) properly localizes to CBs and the nucleoplasm and does not alter CB number when moderately expressed [16], [18]. In contrast, GFP-coilin ON and OFF expression alter normal coilin localization [16]. Specifically, the percentage of cells with only nucleoplasmic coilin and lacking CBs is greatly increased upon ON expression, while OFF expression results in approximately 60% of cells displaying accumulation of this mutant in nucleoli as well as CBs [16]. Transient transfection of these six GFP-tagged coilin constructs into HeLa cells demonstrates that all of them significantly decrease proliferation except for the OFF mutant (Figure 2). These findings show that T122E, ON, S489D and S271/272D act in a dominant negative manner over endogenous coilin to reduce proliferation. Interestingly, cells transiently expressing T122E, S489D or S271/272D do not show any obvious changes in CB number or composition, suggesting that other aspects of coilin function besides a structural role in CB formation may be impacted by the expression of these phosphomimics.

### Generation of inducible coilin phosphomutant cell lines

**Figure 3:**
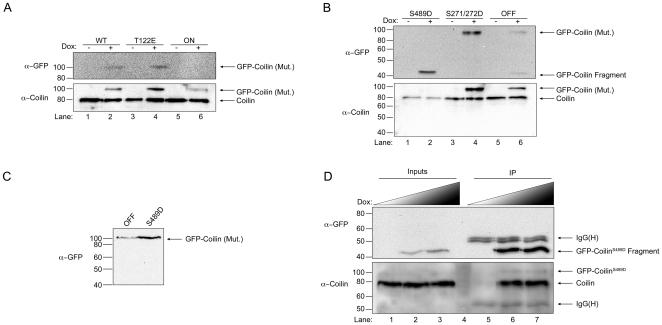
Characterization of doxycycline inducible coilin phosphomutant cell lines. (A and B) Stable cell lines expressing GFP-coilin of GFP-coilin phosphomutant proteins. Non-induced (−) and 24 h doxycycline induced (1 µg/mL, +) cell lysates of GFP-coilin (WT) and GFP-coilin phosphomutants (T122E, ON, S489D, S271/272D or OFF) were subjected to SDS-PAGE, followed by western transfer. The blots were probed with mouse monoclonal anti-GFP antibodies for specific detection of the GFP-tagged coilin proteins (upper panel). The blots were re-probed with rabbit polyclonal anti-coilin antibodies to detect both endogenous coilin and the GFP-tagged WT and coilin phosphomutants (lower panel). Note that S489D and OFF expression generates an approximately 42 kDa degradation product. (C) Transiently transfected GFP-coilin S489D and GFP-coilin OFF phosphomutant proteins do not have a specific degradation product. HeLa cells were transiently transfected with GFP-coilin S489D and GFP-coilin OFF DNA constructs for 24 h followed by lysate generation, SDS-PAGE and western transfer. The blot was probed with antibodies to GFP. (D) Full length GFP-coilin S489D can be detected after doxycycline induction by immunoprecipitation. Doxycycline (0.33 or 1 µg/mL) induced and non-induced GFP-coilin-S489D stable cell extracts were immunoprecipitated with anti-GFP antibodies. The western blot was probed with anti-GFP antibodies for the detection of the GFP-coilin-S489D fragment (upper panel). The same blot was probed with anti-coilin antibodies for endogenous coilin and full length GFP-coilin-S489D protein detection (lower panel). IgG(H) denotes the immunoglobulin heavy chain.

One major difficulty in characterizing the different coilin phosphomutants in regards to CB formation potential and impact on cell proliferation is the presence of endogenous coilin, which can self-interact with the various GFP-coilin proteins and possibly attenuate a given phenotype. To overcome these obstacles, in previous studies we have employed transiently transfected cells in which endogenous coilin is reduced by RNAi [16]. However, since the transiently transfected protein is typically expressed at levels far higher than that found for the endogenous protein, one cannot escape that a given phenotype may be an artifact of overexpression. We therefore generated stable cell lines that can be induced to express a given GFP-coilin protein upon treatment with doxycycline. Each line was at least 60% pure, as assessed by immunofluorescence analysis (discussed below). Western blot analysis demonstrates that the expression of GFP-coilin WT, T122E, and S271/272D proteins is induced by the addition of doxycycline at lower or similar levels to that of the endogenous coilin (Figure 3A and B). In contrast, the relative expression of ON to endogenous coilin is lower in this line compared to the other lines, and it is difficult to detect this protein with antibodies to GFP, although it can be detected with anti-coilin antibodies. Curiously, a full-length GFP-coilin S489D protein could not be detected in lysate after induction. Instead, a fragment of approximately 42 kDa is detected by a GFP antibody but not by a coilin antibody that reacts with the C-terminal 300 amino acids (from amino acid 276–576) (Figure 3B, lane 2). The differential detection of this fragment by GFP antibodies but not by coilin antibodies indicates that the fragment represents GFP (molecular weight 27 kDa) fused to an N-terminal fragment of coilin. This fragment was also observed upon induction of the OFF protein, although full-length protein could be observed for this cell line (Figure 3B, lane 6). Notably, these anti-GFP reacting fragments were not observed in lysate obtained from cells transiently transfected with GFP-coilin S489D and OFF constructs (Figure 3C). This suggests that the more physiological levels of GFP-coilin S489D and OFF found in the inducible cell lines allows for a processing pathway which may be overwhelmed in overexpressing transiently transfected cells. To further confirm that S489D and OFF are subject to a defined degradation pathway, lysate from cells induced to express S489D was subject to immunoprecipitation (IP) with anti-GFP antibodies. In so doing, full-length S489D can be detected in the IP reactions with anti-coilin antibodies (Figure 3D, lanes 6 and 7). Thus the full-length GFP-coilin S489D is made, but quickly processed down to the 42 kDa fragment in the stable cell line. In addition to the small amount of full-length S489D, the GFP antibodies immunoprecipitated the GFP-S489D fragment (lanes 6 and 7, top panel) which co-immunoprecipitated endogenous coilin (lanes 6 and 7, bottom panel).

**Figure 4:**
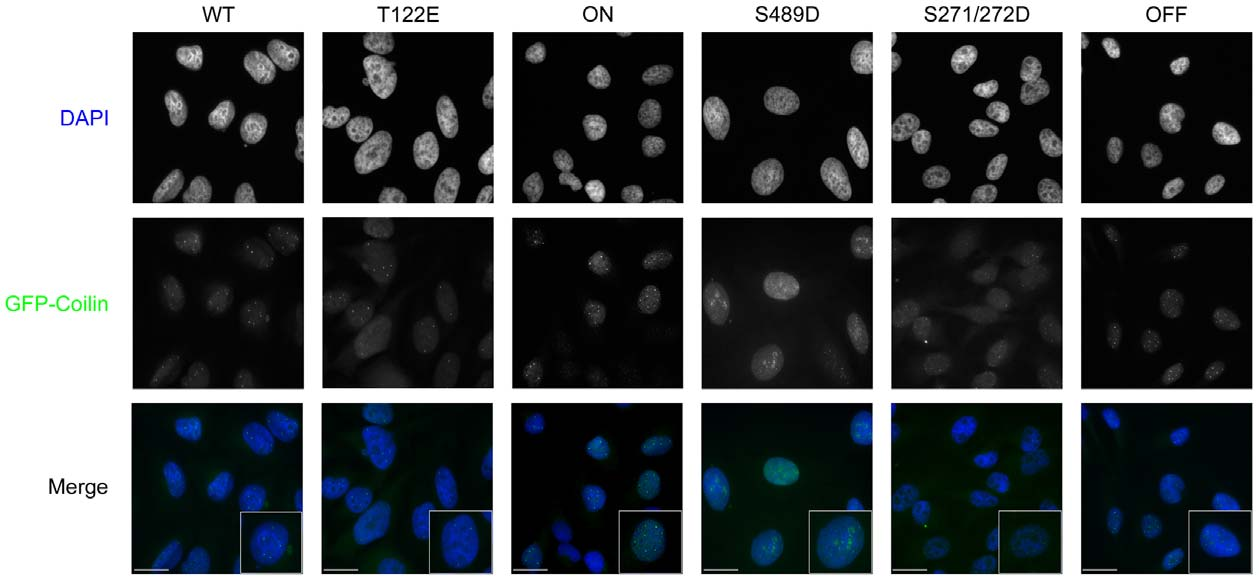
Immunofluorescence analysis of stable cell lines expressing GFP-coilin (WT) or GFP-coilin phosphomutants (T122E, ON, S489D, S271/272D or OFF). Cells were induced for 24 h with doxycycline (1 µg/mL) followed by fixation and extraction. Nuclei were stained with DAPI (blue). DAPI and GFP signal are overlaid in the merge panel. Arrows denote CBs. Double arrowheads mark nucleolar accumulation of S489D and OFF. Triple arrowheads mark small GFP-coilin T122E foci. Scale bars 10 µm.

To further characterize the coilin phosphomutant cell lines, we conducted immunofluorescence analysis and evaluated if the expression of a given coilin mutant would disrupt CBs or alter their number. As shown in Figure 4, the expression of GFP-coilin phosphomutants in the presence of endogenous coilin results in a range of phenotypes. Most notably, the S489D and OFF proteins have accumulations in nucleoli (double arrowhead) and CBs (arrow). In S489D, the majority of expressing cells had this phenotype, while this was observed in approximately 30% of the OFF expressing cells. The other OFF cells had normal coilin localization (in the nucleoplasm and CBs) without nucleolar accumulations. Interestingly, the T122E line has, in addition to normal CBs, dim micro-CB-like structures (triple arrowheads) that were not observed in the other cell lines. Finally, the ON cell line had relatively higher levels of nucleoplasmic coilin compared to the other mutants, but still contained approximately the same percentage of cells with CBs as found for WT.

**Figure 5:**
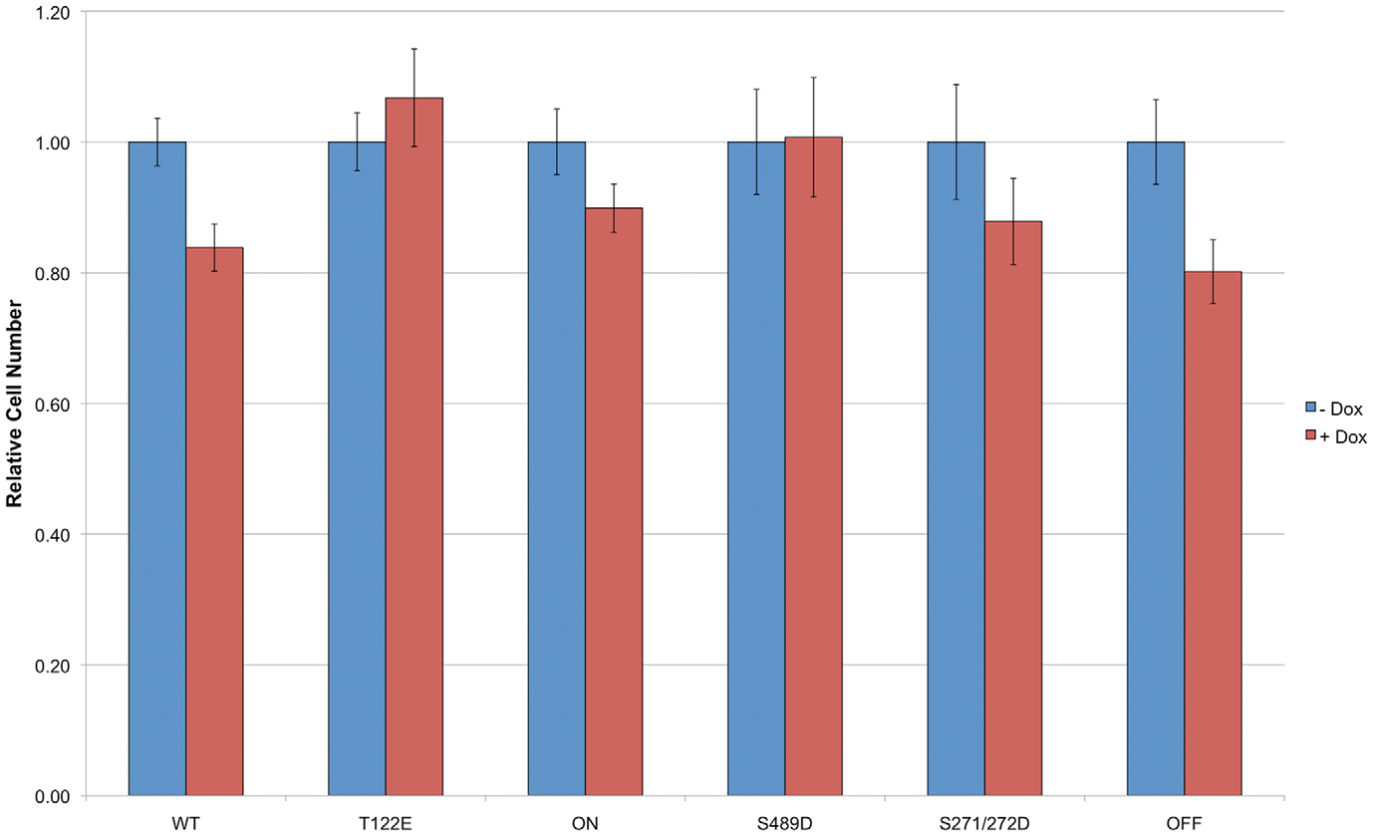
Expression of WT coilin or the OFF coilin mutant decreases cell proliferation in the presence of endogenous coilin. Cell counts were obtained 24 h, 48 h and 72 h after seeding with doxycycline or left untreated. The values for each cell line obtained at 72 h were divided by the value of that line at 24 h. Each line was then normalized to the untreated value for that line. Errors bars denote the percent standard error based on an n of no fewer than 5. There is a significant decrease (p value <0.05) in cell number upon the doxycycline induction of GFP-coilin WT or OFF mutant.

Since we have shown that some transiently transfected coilin phosphomutants impact proliferation, we next investigated if the stable expression of the GFP-coilin mutants likewise altered proliferation rates in the presence of endogenous coilin. Significant decreases in proliferation rates were observed upon induction of GFP-coilin WT and OFF, but were not found in lines expressing the other coilin mutants (Figure 5). However, the presence of endogenous coilin in these experiments may be masking any potential phenotype, so we next set out to analyze the CB formation potential and proliferation rates for the cell lines in a coilin knockdown background.

### Reduction of endogenous coilin in the GFP-coilin phosphomutant cell lines impacts CB number and proliferation rate

**Figure 6:**
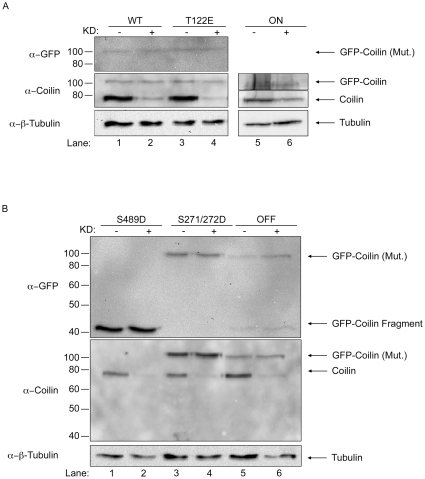
Expression of GFP-coilin and GFP coilin phosphomutant proteins in endogenous coilin depleted stable cell lines. (A and B) Stable cell lines were transfected with coilin siRNA (+) to deplete endogenous coilin, or control siRNA (−). 24 h post siRNA transfection, GFP-coilin or GFP-coilin phosphomutant expression was induced with doxycycline. 24 h later (48 h post siRNA transfection) cells were harvested and lysates were subjected to SDS-PAGE followed by western transfer and probing with anti-GFP antibodies (upper panel). The blots were then probed with anti-coilin antibodies (middle panel) followed by detection of beta-tubulin with anti-beta-tubulin antibodies (lower panel). KD – and + indicates transfection with control siRNA or coilin siRNA, respectively. Note that the GFP-coilin ON signal was too low to be detected with anti-GFP antibodies and could only be detected with anti-coilin antibodies (A, lanes 5 and 6).

To better assess the role of phosphorylation on coilin function in CB assembly, we reduced endogenous coilin by RNAi followed by the induction of the GFP-coilin phosphomutants with doxycycline. This was accomplished by employing a siRNA that targets the 3′ untranslated region (UTR) of the endogenous coilin message. Since the 3′ UTR was deleted in the constructs used to generate the stable cell lines, treatment of cells with this siRNA reduces endogenous coilin but does not affect the expression of the various GFP-coilin proteins (Figure 6). It should be noted, however, that reduction of endogenous coilin and subsequent induction of the respective GFP-coilin protein results in approximately equal amounts of endogenous coilin with the GFP-coilin protein. For example, since the level of GFP-coilin WT upon induction is lower than that of endogenous coilin, knockdown of endogenous coilin results in roughly equal amounts of GFP-coilin WT to endogenous coilin (Figure 6A, lanes 1 and 2). Consequently, any observed changes in CB number and proliferation are indicative of relatively small changes in the ratio of endogenous coilin to the various GFP-coilin proteins, which makes this system more physiologically relevant than that found when conducting transient transfections.

**Figure 7:**
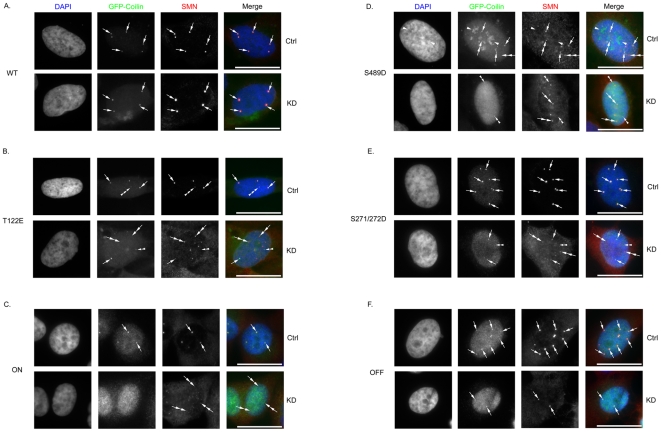
Characterization of coilin phosphomutant localization in endogenous coilin reduced stable cell lines. GFP-coilin and GFP-coilin phosphomutant stable cell lines were transfected with control (Ctrl) or coilin siRNA (KD) for 24 h. 24 h of post siRNA treatment, cells were treated with doxycycline and incubated for another 24 h. The 48 h siRNA transfected and 24 h doxycycline induced stable cell lines were fixed, extracted and immunostained for SMN (red). Nuclei were stained with DAPI (blue). Arrows mark some canonical CBs (containing coilin and SMN). Double arrows mark SMN foci that lack coilin (Gems). Single arrowheads indicate nucleolar coilin accumulation. Double arrowheads mark coilin foci that do not have significant enrichment of SMN, and triple arrowheads indicate dim micro-CB structures. Note that the GFP-coilin ON signal was difficult to detect after the siRNA transfection protocol, so polyclonal GFP antibodies were used to amplify this signal. Scale bars 10 µm.

Immunofluorescence analysis was conducted on each cell line first treated with control or coilin siRNAs and then exposed to doxycycline to induce the expression of the various GFP-tagged coilin proteins. Amongst the different cell lines, only WT is capable of forming robust CBs that contain SMN in most cells upon endogenous coilin knockdown (Figure 7A). The coilin phosphomutants, in contrast, all had reduced CB formation potential in cells treated with coilin-siRNA compared to control-siRNA. Notably, most of the ON expressing cells in the coilin knockdown background were nucleoplasmic and lacked CBs, but contained SMN foci (Gems) (Figure 7C, double arrow). Cell lines that contain numerous CBs upon exposure to control siRNA, such as S271/272D and OFF, showed a significant decrease in CB number after endogenous coilin knockdown (Figure 7E, F). Additionally, the frequency of coilin foci lacking SMN (double arrowhead) and Gems (SMN foci lacking coilin, double arrow) were increased in the S271/272D cell line upon coilin knockdown (Figure 7E). T122E expression in cells treated with coilin siRNA (Figure 7B) generated some CBs (arrow), but also resulted in coilin foci (double arrowhead) that did not efficiently recruit SMN, which accumulated in Gems (double arrow). More drastic changes were observed for S489D upon coilin siRNA treatment (Figure 7D). In control treated cells, most cells have accumulations of this protein in CBs (arrow) and nucleoli (arrowhead), identical to that observed without siRNA treatment after induction (Figure 4). However, in coilin siRNA treated cells, the nucleolar accumulation of S489D is less discrete and the foci formed by GFP-coilin (S489D) (double arrowhead) do not accumulate SMN (double arrow). Alterations in nucleolar accumulations were also observed for OFF, which normally has around 30% of cells showing nucleolar localization. In contrast, like S489D, the accumulation of OFF in nucleoli is reduced upon siRNA treatment (Figure 7F). Foci formed by OFF can recruit SMN, however. Apart from seeing a reduction in CB number upon endogenous coilin knockdown, another predominant phenotype was the disruption of SMN interaction with CBs. Most cells exhibited either lack of SMN accumulation in coilin foci, or the presence of SMN in Gems. Collectively, these results clearly indicate that coilin phosphorylation directly impacts canonical CB formation.

**Figure 8:**
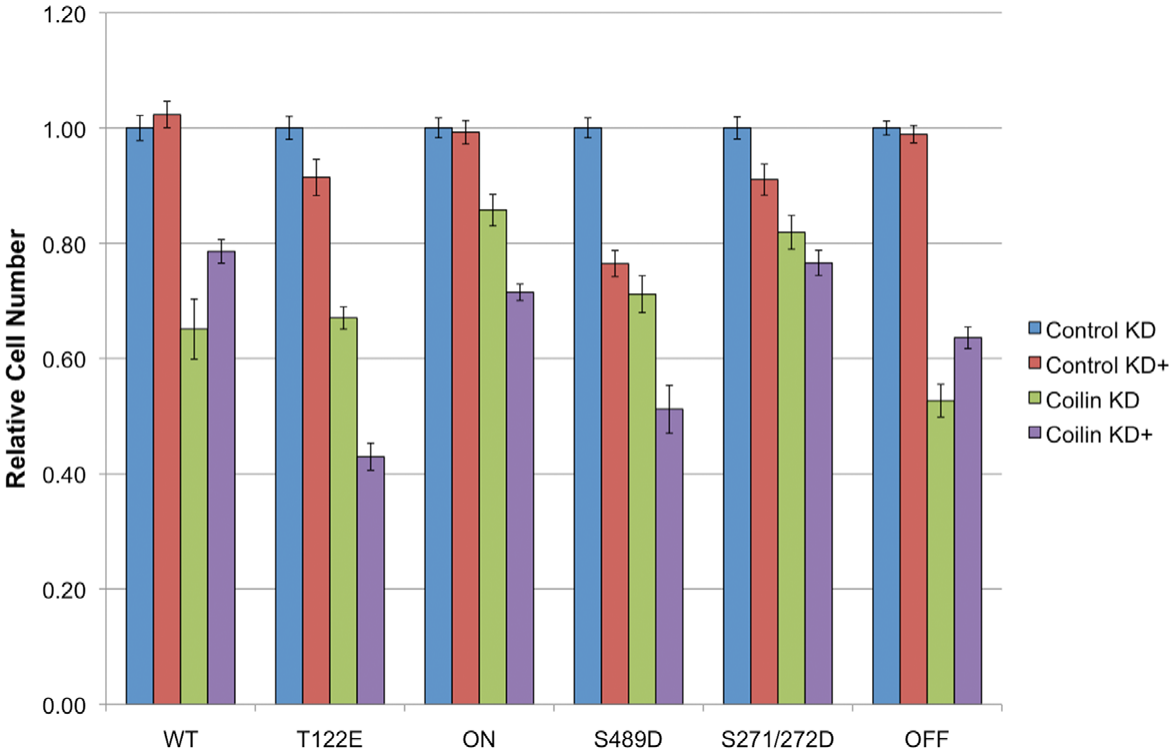
Expression of WT coilin or the OFF coilin mutant rescues reduction in proliferation associated with endogenous coilin depletion. Stable cells untreated or induced to express the various GFP-tagged coilin proteins by doxycycline treatment (+) were transfected with control (control KD) or coilin (coilin KD) siRNA. Cells were seeded into 96-well plates and read 72 h later. This corresponds to 96 h post siRNA transfection. The value obtained for 72 h after seeding was normalized to the value obtained for the untreated control knockdown condition for that cell line. WT clone A2F2 was used for these studies. Errors bars denote the percent standard error based on an n of no fewer than 7. There is a significant difference (p value<0.005) between the coilin KD untreated versus doxycycline treated for all cell lines except S271/272D.

To monitor if the expression of the various GFP-coilin proteins would impact cell proliferation, we examined proliferation rates in induced cells treated with control or coilin siRNA (Figure 8). Previous results have shown that coilin reduction decreases proliferation [14], [15], presumably because of diminished snRNP resources as a consequence of abolished CBs. In agreement with these results, decreased proliferation rates were observed in all lines tested upon treatment with coilin siRNA (coilin KD) relative to control siRNA (control KD). In order to test if the induction of the GFP-coilin phosphomutants would rescue, or exacerbate, the decreased proliferation rate as a result of coilin knockdown, cells were treated with doxycycline. The expression of WT or OFF (coilin KD + Dox condition) could partially rescue the reduction of proliferation caused by the endogenous coilin knockdown (coilin KD condition) (p value<0.005). However, none of the other coilin phosphomutants increased proliferation rates in the coilin KD background. Interestingly, the expression of T122E, ON and S489D significantly decreased rates. These findings demonstrate that mutations that alter coilin phosphorylation impact CB formation and decrease proliferation rates.

## Discussion

In order to understand fully the cellular role of the CB, a more detailed assessment of the factors that regulate its assembly and disassembly is needed. Since many of the proteins that are enriched within the CB are phosphorylated, an important first step in this assessment is a characterization of how phosphorylation of a given CB protein impacts CB formation and activity. Coilin is an ideal protein for these studies since it is phosphorylated and plays a clear role in CB organization. Additionally, previous studies have implicated coilin phosphorylation as a key determinant that influences CB formation and activity. For example, coilin phosphorylation increases during mitosis, when CBs disassemble [17]. This hyperphosphorylation of coilin is associated with reduced coilin self-association [18], thereby leading to the hypothesis that coilin self-association regulated by phosphorylation controls CB organization. Other studies have shown that inhibitors of kinases (olomoucine) and phosphatases (okadaic acid) disrupt CBs and relocate coilin to the nucleolus [32], [33], suggesting that coilin phosphorylation may be altered by these inhibitors. To date, only two kinases (casein kinase II and cyclinE/Cdk2) and one phosphatase (PPM1G) have been shown to modify coilin in vitro [16], [18], [32], but the variety of different kinase motifs present in coilin indicate that many more kinases participate in its modification.

Recent phosphopeptide mapping studies demonstrate that human coilin is phosphorylated on at least 17 residues [4,5,6,7,8, PHOSIDA protein phosphorylation database]. These residues are S105, T122, T265, S271, S272, T277, T303, S305, T456, S486, S489, S566, S568, T570, S571, S572 and T573. Interestingly, these modified residues are grouped in four different regions of coilin and are not dispersed throughout the protein. The first grouping (S105 and T122) lies in the region of coilin that contains the nuclear localization signals and putative nucleolar localization signal. The second grouping (T265, S271, S272, T277, T303 and S305) in the middle of coilin contains many glutamic and aspartic acids. Consequently, phosphorylation of these six residues may further confer a negative charge to this region of coilin, and subsequently impact its folding and interactions. Since the coilin N-terminus and C-terminus are conserved across species, but the middle portion is not, it is possible that these modifications are more paramount in human coilin. The third grouping (T456, S486, S489) lies in the region of coilin that interacts with SMN (the RG box) and Sm proteins, and contains the newly defined tudor domain [19] (Figure 1). The modification of residues within this region may therefore regulate coilin interaction with these proteins. The final grouping (S566, S568, T570, S571, S572 and T573) lies in the very C-terminus of coilin. We have previously showed that the C-terminus of coilin controls CB number [34]. Given the large number of phosphorylated residues in this region, it is reasonable to speculate that this modification is also a determinant in the control of CB number. The impact of these modifications may be more important in human coilin, however, as the very C-terminus is not highly conserved in mouse and frog coilin [34]. Upon consideration of the 11 phosphorylation sites studied here (Figure 1), 7 are conserved in mouse and 4 are conserved in frog, indicating their significance in coilin function across species, but suggesting a higher degree of regulation by this modification in human.

Mutagenesis studies have identified two additional putative phosphorylation sites (S184 and S202) that, when mutated (S184A and S202D), cause coilin to localize in the nucleolus [18], [33]. The S184 site contains a consensus cyclinE/Cdk2 kinase motif, but neither this site, nor the S202 residue, have been shown to be phosphorylated by phosphopeptide analyses. Regardless, the finding that mutation of these residues impacts coilin localization underscores the importance of the non-conserved middle region of human coilin. It is possible that these mutations facilitate the unmasking of a putative nucleolar localization signal (NoLS) located in the N-terminus of coilin (Figure 1). Exposure of this NoLS may also account for the nucleolar accumulation of N-terminal fragments observed in the S489D and OFF stable cell lines (discussed below). A clear understanding of how phosphorylation (or mutation of phosphorylation sites) impacts coilin structure is lacking. Prediction programs, such as Disopred2 (University College London), indicate that coilin contains a large central region (from aa 97–455) that is intrinsically disordered. In addition, the very C-terminus of coilin, which contains 6 phosphoresidues, is also predicted to be intrinsically disordered. In contrast, the N-terminal 96 aa, which contains the coilin self-association domain, and a region from aa 456–563, which contains the coilin tudor domain (Figure 1), are predicted to have structure. Since most of the known phosphorylation sites in human coilin lie within regions of predicted intrinsic disorder, it may be that these modifications alter coilin interaction with other proteins as opposed to altering specific protein folds.

In this study, we have tested different coilin phosphomutants for their ability to form CBs and affect cellular proliferation. These findings are summarized in Tables 1, 2, 3. The residues we have mutated have been shown by phosphopeptide analyses to be phosphorylated (Figure 1) and are found in each of the groupings described above. Studies were conducted using both transiently transfected cells (Figure 2) and stably transfected cell lines engineered to express physiological levels of different coilin phosphomutants upon induction. Transient transfection of GFP-coilin T122E, ON, S489D and S271/272D reduces cell proliferation (Figure 2), suggesting that these mutants act in a dominant negative manner over endogenous coilin when overexpressed.

**Table 1:** Transient expression of various GFP-coilin proteins in the presence of endogenous coilin.

Coilin Protein	Expression	Localization				Proliferation Impact
		CBs	Nucleolar	Nucleoplasmic	Other	
WT	High	95%	--	5%	--	None
T122E	High	95%	--	5%	--	Decrease
ON	High	32%	--	68%	--	Decrease
S489D	High	95%	--	5%	--	Decrease
S271/272D	High	95%	--	5%	--	Decrease
OFF	High	40%	60%	--	--	None

Expression denotes the ratio GFP-coilin or mutants thereof to endogenous coilin.

The data for the localization of WT, ON and OFF taken from [16].

Proliferation impact reflects the change in proliferation rate when compared to the rate of cells expressing WT GFP-coilin.

**Table 2:** Stable inducible expression of various GFP-coilin proteins in the presence of endogenous coilin.

Coilin Protein	Expression	Localization				Proliferation Impact
		CBs	Nucleolar	Nucleoplasmic	Other†	
WT	Low	95%	--	5%	--	Decrease
T122E	Equal	90%	--	10%	30%	None
ON	Low	95%	--	5%	--	None
S489D	N/A	10%	90%	--	--	None
S271/272D	Equal	95%	--	5%	--	None
OFF	Equal	70%	30%	--	--	Decrease

Expression denotes the ratio GFP-coilin or mutants thereof to endogenous coilin.

† Some cells share different combinations of these phenotypes. Therefore, these percentages do not add up to 100. Phenotypes classified as other include SMN foci lacking coilin (Gems), coilin foci lacking SMN, and coilin micro-foci.

N/A  =  not applicable since the S489D protein is degraded in the stable cell line.

Proliferation impact in reflects the change in proliferation rate of a given stable cell line with doxycycline compared to the proliferation rate of the same line in the absence of doxycycline.

**Table 3:** Stable inducible expression of various GFP-coilin proteins with endogenous coilin knockdown.

Coilin Protein	Expression	Localization				Proliferation Impact
		CBs	Nucleolar	Nucleoplasmic	Other†	
WT	Equal	95%	--	5%	10%	Rescue
T122E	Equal	5%	--	95%	90%	Decrease
ON	Low	5%	--	95%	70%	Decrease
S489D	N/A	5%	--	95%	90%	Decrease
S271/272D	Equal	15%	--	85%	90%	None
OFF	Equal	5%	--	95%	50%	Rescue

Expression denotes the ratio GFP-coilin or mutants thereof to endogenous coilin.

N/A  =  not applicable since the S489D protein is degraded in the stable cell line.

† Some cells share different combinations of these phenotypes. Therefore, these percentages do not add up to 100. Phenotypes classified as other include SMN foci lacking coilin (Gems), coilin foci lacking SMN, and coilin micro-foci.

Proliferation impact reflects the change in proliferation rate of a given stable cell line with endogenous coilin knockdown and doxycycline compared to the proliferation rate of the same line with coilin knockdown but no doxycycline. Rescue indicates that the induced protein increased proliferation in the coilin knockdown background, whereas decrease means the induced protein decreased proliferation rates lower than that found with coilin knockdown alone. No change in proliferation rates in the coilin knockdown background upon induction of mutant is denoted as none (as found for the S271/272 mutant).

To reduce the possibility of overexpression artifacts as a consequence of transient transfection, we utilized the doxycycline-induced cell lines in combination with endogenous coilin reduction by RNAi. Several interesting observations were made during the course of these studies using these lines. First, a correlation can be made between the presence of the GFP-coilin fragment observed in the western analysis of S489D and OFF lysates (Figure 3) and nucleolar localization (Figure 4). Indeed, we have previously shown that N-terminal fragments of coilin, comprising of aa 1–248 and 1–315, co-localize with fibrillarin and Nopp140 in CBs and nucleoli [18], similar to the patterns observed here for S489D and OFF. Since lysate obtained from cells transiently transfected with GFP-coilin S489D and GFP-coilin OFF do not have this distinctive degradation product (Figure 3), we conclude that the more physiological expression levels of the mutants found in the stable cell lines allows for a specific degradation event, possibly because these phosphomutants are deleterious to the cell. The identification and regulation of this processing event are current subjects of investigation. It is noteworthy that SMN has been shown to be subjected to specific processing by the protease calpain [35], [36]. Nuclear calpains or related proteases may thus also regulate CB organization by generating specific coilin fragments.

The second major observation made from studies using the coilin phosphomutant cell lines is the inability of these mutants to form canonical CBs in conditions of endogenous coilin depletion (Figure 7). In fact, only GFP-coilin WT is able to consistently form CBs that contain SMN when endogenous coilin is reduced by RNAi. Strikingly, the nucleoplasmic localization (lacking CBs) of ON (Figure 7C) is consistent with hyperphosphorylation of coilin during mitosis leading to CB disassembly. It is also interesting to note that the nucleolar accumulation observed for the S489D and OFF mutants is abrogated in coilin knockdown cells (Figure 7D and F), but the degradation product is still present in this condition as assessed by western blotting (Figure 6B). These findings suggest that overall coilin levels may influence the localization of the S489D and OFF phosphomutants. The final observation made from these stable cell lines is the correlation between CB formation in conditions of endogenous coilin depletion and rescue of the reduction of proliferation caused by coilin knockdown (Figure 8). Previous work has shown that coilin reduction diminishes cell proliferation [14], [15], and this was observed in all six of the doxycycline inducible stable cell lines. However, amongst the different coilin proteins, only GFP-coilin WT and OFF expression significantly increased proliferation above that obtained with coilin knockdown. Curiously, the similar results obtained for WT and OFF in each of the proliferation studies conducted here (Figures 2, 5 and 8), suggest that the OFF mutant can best reproduce the function of WT coilin that impacts proliferation. In contrast to WT and OFF, none of the other phosphomutants could rescue proliferation rates above that found with endogenous coilin knockdown, but instead result in no change (S271/272D) or a significant decrease (T122E, ON, S489D) (Figure 8).

By employing stable cell lines that can be induced to express, at near physiological levels, coilin phosphomutants, we demonstrate here the importance of various coilin phosphoresidues in the formation of canonical CBs. These lines, along with others in development, provide a valuable resource towards understanding how phosphorylation regulates CB assembly, activity and disassembly.

## Materials and Methods

### Cell culture, DNA transfection and DNA constructs

HeLa cells were obtained from the American Type Culture Collection (Manassas, VA, USA) and cultured as previously described [37]. Transient transfections of plasmid DNA were performed using Fugene 6 (Promega, Madison, WI, USA) according to the manufacturer's protocol. A previously described GFP-coilin construct [18] was used as a template for Quick Change Mutagenesis (Stratagene, Santa Clara, CA, USA) to generate all the phosphomutants in this study. The ON and OFF GFP-coilin mutants have been described previously [16]. In order to clone the GFP-coilin (or mutants thereof) cDNA into the pLVX-Tight-Puro vector (Clontech, Mountain View, CA, USA) for subsequent lentiviral generation, a Not I restriction site was introduced upstream of the GFP and an Mlu I was introduced downstream of the coilin coding sequence, also using the Quick Change Mutagenesis procedure. The Not I and Mlu I digested GFP tagged coilin phosphomutants were cloned into pLVX-Tight-Puro vector cut with the same enzymes and sequence verified.

### Preparation of lentivirus, transduction and generation of coilin phosphomutant stable cell lines

Generation of VSV-G-pseudotyped lentivirus vectors were performed by co-transfecting three plasmids: the vector containing the gene of interest, VSV-G-expressing construct (pVSV-G), and the packaging construct pCMVΔR 8.9, which carries *gag*, *pol*, *tat*, and *rev* genes. These constructs were transiently co-transfected into HEK293FT (Invitrogen, Carlsbad, CA, USA) cells at a 3∶2∶1 mass ratio by using either TransIT-293 (Mirus, Madison, WI, USA) or Fugene 6 (Promega, Madison, WI, USA) transfection reagent according to the manufacturer's protocol. After 4–6 h incubation, the medium was replaced with fresh culture medium and left to incubate. The lentiviral supernatants were collected 48 h post transfection. The viral supernatant was centrifuged for 5 min at 500 g at 4°C, followed by filtering with a 0.4 µm polyethersulfone filter (Nalgene, Rochester, NY, USA). Six different lentiviral supernatants were generated: GFP-coilin WT, GFP-coilin T122E, GFP-coilin ON (11 residues shown in figure 1 were converted to aspartic acid (for serine) or glutamic acid (for threonine), GFP-coilin S489D, GFP-coilin S271/272D, GFP-coilin OFF (11 residues shown in figure 1 were converted to alanine). HeLa Tet-On cells (Clontech, Mountain View, CA, USA) were transduced with a 1∶1 dilution of viral supernatant and fresh medium containing 8 µg/ml of polybrene (Sigma, St. Louis, MO, USA), and incubated for 6 h. The cells were then trypsinized and plated at 1∶29 and 1∶2 dilutions into 150 mm^2^ dishes. The cells were cultured with DMEM medium (Mediatech, Manassas, VA, USA) containing 10% fetal bovine serum (HyClone Laboratories, Inc., Logan, UT, USA), 100 µg/mL penicillin/streptomycin (Mediatech, Manassas, VA, USA), and 100 µg/ml G418 Sulfate (Gibco, Carlsbad, CA, USA) in a 5% CO_2_ incubator at 37°C. After 48 h transduction, the cells were selected by adding 1 µg/ml puromycin (InvivoGen, San Diego, CA, USA) to the culture medium. After two weeks selection, distinct colonies were picked and transferred to 6-well plates. The clones were induced for 24 h with 1 µg/mL doxycycline (Clontech, Mountain View, CA, USA) to verify GFP-coilin or phosphomutant expression as assessed by western blot and immunofluorescence. The expressing clones were sub-cloned to increase purity by seeding into 96-well plates, which were then verified for protein expression as previously described. At least two clones were obtained for each lentiviral construct and verified to share similar phenotypes. Representative clones were then used for the remaining studies. These clones are: WT (A3B8 and A2F2), T122E (B5F9), ON (A6), S489D (A4B5), S271/272D (B1F5), OFF (A6).

### Antibodies, immunofluorescence, immunoprecipitation and western blotting

Mouse monoclonal antibodies against GFP were purchased from Roche (Mannheim, Germany) and used at a dilution of 1∶250 for detection on western blots and 4 µg for immunoprecipitation reactions. Rabbit polyclonal antibodies against GFP were obtained from Abcam (Cambridge, MA, USA) and used at a dilution of 1∶800 for immunofluorescence detection of the GFP-coilin ON protein after siRNA treatment. Mouse monoclonal antibodies against β-tubulin were used at a dilution of 1∶1000 (western) and purchased from Sigma (St. Louis, MO, USA). Rabbit polyclonal antibodies against coilin (H300) were purchased from Santa Cruz Biotechnology (Santa Cruz, CA, USA) and used at dilutions of 1∶500 (western) and 1∶200 (immunofluorescence). Mouse monoclonal antibodies against SMN were acquired from BD Transduction Laboratories (San Jose, CA, USA) and used at a dilution of 1∶100 for immunofluorescence. Protocols for immunofluorescence, image acquisition and western blotting were conducted as described previously [37]. Cell lysate generation and immunoprecipitations were performed as previously described [16], except RIPA buffer (50 mM Tris-HCl pH 7.6, 150 mM NaCl, 1% NP-40, 0.25% sodium deoxycholate, 0.1% SDS, 1 mM EDTA) was used.

### RNA interference and cell proliferation

Reduction of endogenous coilin message was accomplished using a siRNA that targets the 3′ untranslated region (UTR) of coilin, obtained from Integrated DNA Technology (Coralville, IA, USA). The 3′ UTR of coilin has been deleted in the GFP-coilin WT and phosphomutant constructs, allowing for the specific knockdown of the endogenous message. The non-targeting siRNA#2 (Cat no. D-001210-02) was obtained from Thermo Scientific (LaFayette, CO, USA). Lipofectamine 2000 (Invitrogen, Carlsbad, CA, USA) was used to introduce the coilin and control siRNAs into cells according to the manufacturer's directions.

Proliferation assays were performed using the cell titer blue reagent from Promega (Madison, WI, USA) according to the manufacturer. For proliferation assays employing transient transfections, cells were transfected with the various GFP-tagged coilin constructs as described above. 24 h after transfection, 5000 cells per well of a 96-well dish (24 wells per construct) were seeded. The fluorescence was read 24 h, 48 h and 72 h after seeding with a FLx800 Spectrophotometer (BioTek, Winooski, VT, USA) using a 490/540 filter set. The readings obtained from 72 h were divided by the readings obtained from the 24 h time point, and all values were normalized to WT. For proliferation assays using the stable cell lines in the presence of endogenous coilin, 5000 cells per well of a 96-well dish were seeded in the presence of 1 ug/ml doxycycline to induce expression of the various GFP-coilin proteins or left untreated. Plates were then read 24 h, 48 h, and 72 h after seeding. The values from the 72 h reading were divided by the 24 h reading, and each line was normalized to the untreated (no doxycycline) condition for that cell line. For proliferation assays using the stable cell lines and siRNA transfection, cells were seeded in the presence of 1 µg/ml doxycycline to induce expression or left untreated. 18 h later, doxycycline treated and untreated cells were transfected with control or coilin siRNA. 24 h post-transfection, 5000 cells per well of a 96-well dish were seeded. Doxycycline was added during seeding at a concentration of 0.2 µg/ml. The fluorescence was read 72 h after seeding (96 h post-transfection). The readings obtained from 72 h after seeding were normalized to the value obtained for the untreated control knockdown condition for that cell line.
